# Two elderly patients with normal creatinine and elevated cystatin C – a case report

**DOI:** 10.1186/s12882-017-0508-7

**Published:** 2017-03-14

**Authors:** Amina Loesment-Wendelmuth, Elke Schaeffner, Natalie Ebert

**Affiliations:** 1 Charité - Universitätsmedizin Berlin, Augustenburger Platz 1, 13353 Berlin, Germany; 2 Institute of Public, Charité - Universitätsmedizin Berlin, Seestrasse 73, Haus 10, D-13347 Berlin, Germany

**Keywords:** Kidney function, Elderly, Creatinine, Cystatin C, GFR equation, Non-GFR determinants, Case report

## Abstract

**Background:**

Serum creatinine concentration (Scr) and creatinine based GFR estimating equations (eGFR_cr_) are commonly used as an estimate of GFR. However, serum creatinine concentration is also influenced by non-GFR determinants. This case report presents two elderly patients with normal Scr but elevated serum cystatin C concentration (Scys) where the exclusive assessment of Scr would have lead to an overestimation of GFR and would have misclassified the patients as having a normal kidney function.

**Case presentation:**

Patient 1, a 102-year-old woman, presented with a Scr of 0.45 mg/dl, while her Scys was elevated (1.55 mg/l). Depending on which of the five GFR estimating equations was used, the patient could be classified into four different CKD-Stages (2, 3a, 3b and 4). The largest difference between the eGFR-results was 94 ml/min/1.73 m^2^ (Δ-eGFR).

Patient 2, an 88-year-old man, also had normal Scr (0.93 mg/dl) but elevated Scys (1.55 mg/l). An iohexol clearance measurement yielded a measured GFR (mGFR) of 44 ml/min/1.73 m^2^. Four out of five GFR equations would have overestimated the patient’s kidney function.

**Conclusion:**

The presented cases highlight the influence of non-GFR determinants on Scr and demonstrate the variability of eGFR results depending on the filtration marker and GFR equation used. Especially for older adults, it shows the great clinical importance of understanding the limitations of each filtration marker and of identifying situations in which relying on eGFR_cr_ alone can lead to false estimation of kidney function. In these situations, cysC based GFR equations may provide improved accuracy of GFR assessment and may protect patients from drug overdosing and the abundant use of contrast agents.

## Background

Generally, the serum concentration of an endogenous filtration marker, such as creatinine, is used as a rough estimate of the glomerular filtration rate (GFR) and is also the central element of all GFR estimating equations. In an equilibrium of endogenous production and renal elimination, creatinine correlates inversely with GFR. Its serum concentration however, can be influenced by non-GFR determinants such as muscle mass. Cystatin C is another endogenous filtration marker but only very rarely routinely monitored. This case report presents two elderly patients with perfectly normal serum creatinine concentration (Scr), but clearly elevated serum cystatin C concentration (Scys). The exclusive assessment of creatinine would have most probably misclassified these patients as being “kidney-healthy”. Thus, it highlights the importance of putting serum creatinine levels into the individual patient context and of identifying situations in which further assessment of kidney function, using ScysC or even direct measurement of GFR (mGFR), is needed.

## Case presentation

A 102-year-old woman (patient 1) took part in the Berlin Initiative Study (BIS), a large prospective population-based cohort study focusing on the epidemiology of chronic kidney disease in older adults. Her only pre-existing condition was a peripheral artery disease. Permanent medication included pantoprazole and novaminsulfon on a regular basis and magnesium as needed. The physical examination yielded a height of 156 cm and weight of 42 kg corresponding to a body mass index (BMI) of 17 kg/m^2^. Her resting blood pressure and pulse were 151/69 mmHg and 78/min, respectively. The patient’s lab results revealed haemoglobin 10.6 g/dl, CRP 1.28 mg/l, urea 28 mg/dl, albumin 30.2 g/l, calcium 2.27 mmol/l, phosphate 1.12 mmol/l. Albumin-creatinine-ratio (ACR) was below 30 mg/g.

**Fig. 1:**
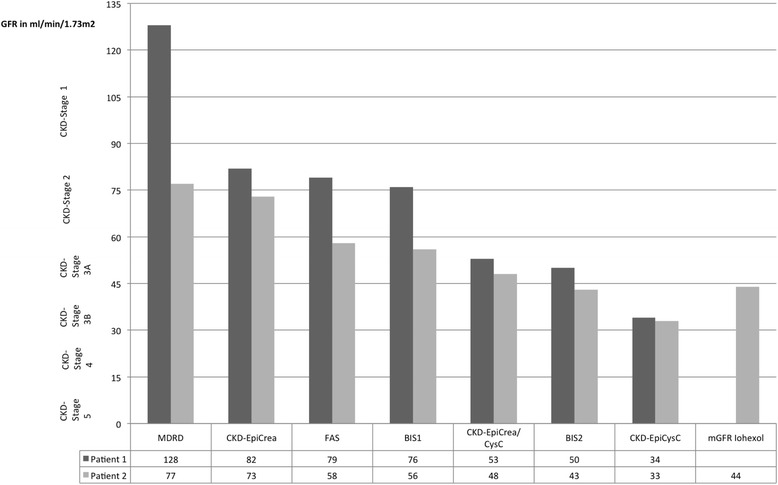
Estimated GFR results depending on different equations in patient 1 and 2

At presentation, Scr was within the normal range (0.45 mg/dl), while Scys was elevated (1.55 mg/l). GFR estimating equations (eGFR) yielded the following results: MDRD [1]: 128 ml/min/1.73 m^2^, CKD-Epi_cr_ [2]: 82 ml/min/1.73 m^2^, FAS [3]: 79 ml/min/1.73 m^2^, BIS1 [4]: 76 ml/min/1.73 m^2^, CKD-Epi_cr/cys_ [5]: 53 ml/min/1.73 m^2^, BIS2 [4]: 50 ml/min/1.73 m^2^, and CKD-Epi_cys_ [5]: 34 ml/min/1.73 m^2^. The largest difference between creatinine-based eGFR-results (Δ-eGFR_cr_) was 49, between all equations 94 ml/min/1.73 m^2^. Thus, based on eGFR results the patient could at the same time be classified into four different CKD-stages (2, 3a, 3b and 4), depending on the GFR estimating equation used (see Fig. 1).

Patient 2 was an 88-year old male participant of the BIS study. Beside a diagnosis of arterial hypertension, he had no history of further chronic illnesses. A beta-blocker was his only permanent daily medication. His height was 167 cm and he weighed 69 kg (BMI 25 kg/m^2^). His resting blood pressure was 213/89 mmHg, his heart rate 89 bpm. Blood analysis yielded the following results: haemoglobin 13.1 g/dl, CRP 0.61 mg/l, urea 43 mg/dl, albumin 35.5 g/l, calcium 1.98 mmol/l, phosphate 0.98 mmol/l. ACR was below 30 mg/g.

Similar to the first patient, his Scr was within the normal range (0.93 mg/dl), while his Scys was elevated to 1.77 mg/l. This corresponded to the following eGFR-results: MDRD: 77 ml/min/1.73 m^2^, CKD-Epi_cr_: 73 ml/min/1.73 m^2^, FAS: 58 ml/min/1.73 m^2^, BIS1: 56 ml/min/1.73 m^2^, CKD-Epi_cr/cys_: 48 ml/min/1.73 m^2^, BIS2: 43 ml/min/1.73 m^2^, CKD-Epi_cys_: 33 ml/min/1.73 m^2^. The largest difference between eGFR-results was 40 ml/min/1.73 m^2^ (Δ-eGFR). This patient belonged to the BIS subgroup in which measurement of GFR using iohexol plasma clearance had been conducted. His measured GFR (mGFR) was 44 ml/min/1.73 m^2^, which demonstrated that four out of five eGFR-equations would have overestimated his kidney function (see Fig. 1).

When analyzing the population of the Berlin Initiative Study (*n* = 2096, mean age 80.4 years) 607 participants showed the combination of a normal Scr concentration (men: <1.17 mg/dl; women: <0.95 mg/dl) and an elevated Scys level (men and women: >0,96 mg/l). This resulted in 29% of the BIS participants being classified as having a normal kidney function in case only Scr had been taken into consideration.

## Discussion

Scr has been abundantly used for the assessment of GFR for almost 100 years. However, glomerular filtration is only one of several variables that determine creatinine concentration. In certain clinical situations, relying on creatinine based GFR estimating equations (eGFR_cr_) alone can lead to false estimation of kidney function. The presented cases demonstrate impressively the strong influence of non-GFR determinants on creatinine concentration. In addition, they highlight the variability of eGFR results depending on (1) the filtration marker and (2) eGFR equation used. It is thus of great clinical importance to understand the value and shortcomings of each filtration marker to be able to identify situations in which certain markers, in this case eGFR_cr_, are less accurate [6].

For the described patients, low creatinine generation due to low muscular mass was certainly the most influential non-GFR determinant. Low muscular mass is common in the elderly due to muscle wasting and malnutrition, being accompanied by a decreased serum creatinine, which in turn leads to an overestimation of kidney function. In our cohort study, which exclusively consists of individuals over 70 years, one third of these participants showed the combination of a normal Scr with an elevated Scys. Against the background of demographic aging clinicians should be aware of the effect of a diminishing muscular mass on serum creatinine concentration.

Other non-GFR determinants that cause a decline in serum creatinine are a protein- and meat-reduced diet, increased excretion by tubular secretion or gastrointestinal elimination [7] as well as increased extracellular fluid [8]. On the other hand, certain medication (trimethoprim, cimetidin, fenofibrat) inhibit tubular secretion of creatinine or decrease extrarenal elimination by inhibition of the gut creatinase (certain antibiotics), resulting in higher serum creatinine levels and thus leading to an underestimation of kidney function [9].

In certain situations (i.e. age ≥70 years, malnutrition, liver cirrhosis, edema, diabetic hyperfiltration), Scys based GFR estimating equations (eGFR_cys_) may therefore provide an improved accuracy of GFR assessment and CKD-classification. Scys is produced at a constant rate in all nucleated cells [10] and in contrast to creatinine its rate of production is independent of muscular mass and gender. Determining Scys in the two cases described above will thus protect patients from drug overdosing and from the abundant use of contrast agents.

There are however non-GFR determinants that influence Scys such as inflammation, obesity (probably due to proinflammatory properties of adipocytes) autoimmune disease, hyperthyreosis, malignoma and nicotine consumption [11]. Some of them are very common in older age as well (i.e. inflammation). Even more, there are studies that suggest that Scys might increase with age, which would in turn lead to an underestimation of GFR in older adults [12, 13]. Unfortunately we had no mGFR in patient 1 which would have provided a definite GFR value given the fact that patient 1 also had slightly increased CRP suggesting mild inflammation.

In conclusion, especially in older adults clinicians should appreciate the limitations of endogenous filtration markers. Their awareness needs to be sharpened to take individual patient characteristics (in our cases especially cachexia, muscle wasting, malnutrition) into consideration in order to identify constellations where creatinine is insufficient. In such situations cystatin C-based eGFR equations should be preferred. In other situations, i.e. states of inflammation Scys might not be the ideal marker. Thus, in older vulnerable adults where sometimes crucial decisions have to me made such as dosage of nephrotoxic drugs or the eligibility of kidney donation, direct clearance measurement should be considered as a reasonable alternative since the accurate determination of renal function can be vitally important.

## Conclusion

The presented case report demonstrates impressively the strong influence of non-GFR determinants on creatinine concentration and highlights that the choice of renal markers for assessing kidney function should be considered very cautiously, especially in older adults. Also, it indicates circumstances where cystatin C may be a superior biomarker for clinical decision-making and refers to situations where measured GFR should be considered.

## Abbreviations

ACR: Albumin-creatinine-ratio
BIS: Berlin initiative study
BMI: Body mass index
eGFR: Estimated GFR
mGFR: Measured GFR
Scr: Serum creatinine concentration
Scys: Serum cystatin C concentration
